# Low-Toxicity Solvents for the Extraction of Valuable Lipid Compounds from Octopus (*Octopus vulgaris*) Waste

**DOI:** 10.3390/foods12193631

**Published:** 2023-09-30

**Authors:** Lucía Méndez, Alicia Rodríguez, Santiago P. Aubourg, Isabel Medina

**Affiliations:** 1 Department of Food Technology, Marine Research Institute (CSIC), 36208 Vigo, Spain; luciamendez@iim.csic.es (L.M.); medina@iim.csic.es (I.M.); 2 Department of Food Science and Chemical Technology, Faculty of Chemical and Pharmaceutical Sciences, University of Chile, Santiago 8380494, Chile; arodrigm@uchile.cl

**Keywords:** octopus, by-products, low-toxicity solvents, simplex-lattice design, optimization, phospholipids, EPA, DHA, polyunsaturated/saturated, ω3/ω6 ratio

## Abstract

This study focused on the recovery of valuable lipid compounds from octopus (*Octopus vulgaris*) by-products. Extraction conditions of total lipids (TLs), phospholipids (PLs), eicosapentaenoic acid (EPA), and docosahexaenoic acid (DHA) were optimized by employing a Simplex-Lattice design; for it, different relative concentrations of three low-toxicity solvents (ethanol, acetone, and ethyl acetate) were considered. The optimization process was also addressed in reference to fatty acid (FA) ratios (total polyunsaturated FAs/total saturated FAs and total ω3 FAs/total ω6 FAs). The variance analysis of multiple regression data demonstrated that the quadratic model was significant (*p* < 0.05) for TL, PL, and DHA values and the ω3/ω6 ratio. As a result, the following optimized values were obtained: 113.8 g·kg^−1^ dry by-products (TLs), 217.3 g·kg^−1^ lipids (PLs), 22.55 g·100 g^−1^ total FAs (DHA), and 3.70 (ω3/ω6 ratio). According to the model developed, optimized values were shown to correspond to the following relative solvent concentrations (ethanol/acetone/ethyl acetate): 0.46/0.00/0.54, 0.93/0.07/0.00, 0.83/0.17/0.00, and 0.64/0.00/0.36, respectively. Comparison to yields obtained by the conventional chloroform/methanol method was carried out. A novel strategy based on the employment of low-toxicity solvents is proposed for the extraction of valuable lipid constituents from octopus waste. A different solvent ratio would be necessary according to the lipid compound concerned.

## 1. Introduction

Fish and invertebrate marine species play an important role in the feeding and development of human society. However, only 50 to 60% of the total catch is used for direct human consumption; thus, seafood processing is considered to be one of the main sources of by-products and leads to important environmental concerns [1,2]. Notably, seafood by-products can be considered an important source of major constituents like lipids, proteins, minerals, and vitamins, in addition to minor components such as pigments, chitin, enzymes, amino acids, and collagen [3,4].

To obtain fats and oils from marine by-products, different kinds of green or low-toxicity methods have been used, such as wet pressing [5], ensilage [6], supercritical fluid extraction [7,8], urea concentration [9], and enzymatic hydrolysis [10]. Among them, great attention has been given to the employment of low-toxicity solvents for lipid extraction. In such studies, solvents have shown valuable possibilities according to their polarity and other physicochemical properties and the concrete substrate to be extracted. Remarkably, such efforts have especially focused on agricultural by-products and have employed solvents like ethyl acetate, glycerol, ethanol, and acetone [11,12]. Concerning seafood, previous studies on the use of low-toxicity solvents cannot be considered abundant. Gigliotti et al. [13] tested acetone/ethanol mixtures for the lipid extraction from Antarctic krill (*Euphausia superba*). Bian et al. [14] maximized the lipid and fatty acid (FA) yield from the microalga *Scenedesmus obliquus* by using subcritical *n*-hexane/isopropanol extraction. Li et al. [15] tested different combinations of low-toxicity solvents for obtaining antioxidants (i.e., vitamin E) and edible oils from the microalga *Scenedesmus dimorphus*. Recently, ethanol, acetone, and ethyl acetate were shown to be alternative solvents for the extraction of valuable lipid compounds such as phospholipids (PLs), ω3 FAs [16], and tocopherols [17] from squid (*Doryteuthis gahi*) waste. In addition to being recommended for green procedures [18], these three solvents provide valuable physicochemical properties such as relative polarity (0.654, 0.355, and 0.228, respectively) and dielectric constant (24, 21, and 6, respectively) [19].

Octopus species constitute highly nutritional seafood that are commercialized in a great variety of products. However, octopus processing leads to the formation of a wide range of by-products (ca. 10–15% of total weight) having high functional value and showing an attracting potential for the integrated exploitation and use of valuable molecules [20,21]. In a seasonal study, octopus (*Octopus vulgaris*) ovaries and digestive glands showed valuable levels of PLs and ω3 FAs [22]. Additionally, juice liquor resulting from industrial giant Pacific octopus (*Enteroctopus dofleini*) processing showed remarkable antihypertensive and antioxidant effects [23] and a high content of polyphenols [24]. Recently, the presence of octopus (*O. vulgaris*) cooking liquor in the packing medium employed during fish canning led to an increased rancidity stability of canned horse mackerel (*Trachurus trachurus*) [25].

The present research focuses on the extraction of valuable lipid molecules from by-products obtained from octopus (*O. vulgaris*) processing. By-products, considered as a single substrate, were extracted with low-toxicity solvents. A comparison to the yields obtained from the conventional procedure (i.e., chloroform/methanol, 1:1) was carried out. A Simplex-Lattice design was developed to optimize the content of total lipids (TLs), total PLs, eicosapentaenoic acid (EPA), and docosahexaenoic acid (DHA), as well as the total polyunsaturated FA (PUFA)/total saturated FA (STFA) and total ω3 FA/total ω6 FA ratios. Based on an advanced mathematical design, a new low-toxicity procedure is presented for the extraction of valuable lipid compounds from octopus by-products.

## 2. Materials and Methods

### 2.1. Initial Octopus By-Products: Analysis and Preparation for Extraction

Octopus (*O. vulgaris*) specimens were captured near the Galician coast (north-west Spain). The corresponding by-products were supplied by Frigoríficos Rosa de los Vientos S. L. (Marín, Pontevedra, Spain). After commercial scission of tentacles and heads, the different kinds of by-products were pooled together, kept in refrigerated conditions (4 °C), and transported (for 30 min) to our laboratory.

In order to analyze the initial by-products, 500 g of this substrate were taken and homogenized. Then, 4 × 10 g homogenized portions (*n* = 4) were separated. In each portion, moisture was determined as the weight difference (1–2 g) before and after 4 h at 105 °C in agreement with the official method 950.46B [26]; results were calculated as g·kg^−1^ by-products. Additionally, conventional lipid extraction was carried out by the Bligh and Dyer [27] method; this procedure employs a single-phase solubilization of the lipids using a chloroform/methanol (1/1) mixture.

A total of 2 × 1 kg of by-products were lyophilized (–70 °C, 72 h, 0.05 mTorr) (Model FD8515-C60, Ilshin Biobase Europe, Ede, The Netherlands). After that, the lyophilized samples were pooled together, minced, and used for the extraction of the lipid fraction.

### 2.2. Description of the Experimental Design Developed

A Simplex-Lattice mixture design was developed, with three experimental factors (relative proportion of each low-toxicity solvent; independent variables) and six response variables (TL, PL, EPA, and DHA contents and PUFA/STFA and ω3/ω6 ratios; dependent variables). This design consisted of fourteen experiments with six different experimental conditions (different low-toxicity solvent ratios) that were carried out in duplicate, as well as two centroids with the same concentration for each of the low-toxicity solvents used.

The extracting conditions applied to lyophilized squid by-products are depicted in Table 1, where the relative content of each solvent is indicated for each extracting condition (0.00–1.00 range). Each of the extracting conditions (from EC-1 to EC-14) was carried out in duplicate.

### 2.3. Lipid Extraction by Low-Toxicity and Conventional Solvents

The following procedure was carried out for the lipid extraction of lyophilized by-products with low-toxicity solvents: 3.5 g of lyophilized by-products and 10 mL of the solvent mixture were stirred for 1 min at 4 °C, centrifuged at 3500× *g* for 10 min at 4 °C, and the supernatant collected. This procedure was repeated two more times and all supernatants were collected together. Then, partial evaporation of solvents was carried out (rotary evaporator; 10 min at 30 °C), and the resulting extract was brought up to a 15 mL volume and was stored at −40 °C before analysis.

The lipid extraction of lyophilized by-products by the conventional method was carried out by the above-mentioned method [27]. Both for conventional and low-toxicity solvent extracts, lipid quantification was expressed as g·kg^−1^ dry by-products.

### 2.4. Lipid Extract Analysis

The total PL content in the lipid extract was measured spectrophotometrically (710 nm; Beckman Coulter DU640 spectrophotometer, Brea, CA, USA) in agreement with the method of Raheja et al. [28]. This method is based on a complex formation between PLs and ammonium molybdate. Results were expressed as g·kg^−1^ lipid extracts.

Acid-catalyzed esterification and transesterification with acetyl chloride in methanol was used for converting lipid extracts into FA methyl esters (FAMEs). The resulting FAMEs were then subjected to gas–liquid chromatography (Perkin Elmer 8700 chromatograph, Madrid, Spain) analysis in order to achieve the separation of FAMEs as shown previously [16,29]. A fused silica capillary column SP-2330 (0.25 mm i.d. × 30 m, Supelco Inc., Bellefonte, PA, USA) was used, programmed from 145 °C to 190 °C at 1.0 °C·min^−1^, from 190 °C to 210 °C at 5.0 °C·min^−1^, held for 13.5 min at 210 °C, and from 210 °C to 230 °C at 5.0 °C·min^−1^. Nitrogen at 10 psi as a carrier gas and flame ionization detector (FID) at 250 °C were employed. For qualitative purpose, the FA retention times were compared to those of standard mixtures (Qualmix Fish, Larodan, Malmo, Sweden; FAME Mix, Supelco, Inc., Bellefonte, PA, USA). For quantitative purposes, peak areas were automatically integrated and C19:0 FA was employed as internal standard. The content of each FA was calculated as g·100 g^−1^ of total FAs.

Results concerning FA groups (STFAs; monounsaturated FAs, MUFAs; PUFAs; ω3 and ω6 FAs) and FA ratios (total PUFAs/total STFAs and total ω3/total ω6) were calculated taking into account the results obtained in individual FAs.

### 2.5. Statistical Analysis

One-way ANOVA (*p* < 0.05) was applied to the data (*n* = 4) obtained from the different lipid composition analyses (total lipid, total PL, EPA, and DHA contents; PUFA/STFA and ω3/ω6 ratios) to investigate differences among the different lipid extracts (low-toxicity and conventional systems) (Statistica version 6.0, 2001; Statsoft Inc., Chicago, IL, USA). Comparison of means was performed using a least-squares difference (LSD) method.

A statistical analytical system was used for multiple regression analysis, ANOVA, canonical analysis, and analysis of ridge maximum of data in the response surface regression (RSREG) procedure. Estimated response surfaces and contours of the estimated response surface were developed using the fitted quadratic polynomial equations obtained from RSREG analysis and holding the process variables with the least effect on the response at a constant value while changing the levels of the other two variables [8]. The 95% confidence intervals of each lipid parameter were calculated, considering the number of replicates and the standard deviation of each sample.

The interaction of the three low-toxicity solvents tested was analyzed by employing an approach based on experiments with mixtures [30]. For this purpose, a {3,2} Simplex-Lattice mixture design was found suitable [31], where the first number in the bracket denotes the number of solvents, and the second number denotes a second-degree model that was employed to estimate the model parameters. Thus, the Scheffé second-degree model [32] was found suitable for this purpose and can be described in agreement with the following equation (Equation (1)):

Y = β_1_X_1_ + β_2_X_2_ + β_3_X_3_ + β_1,2_X_1_X_2_ + β_1,3_X_1_X_3_ + β_2,3_X_2_X_3_ (1)

where Y = response variable; β_i_ = coefficient parameter of each single solvent (linear terms); β_i,j_ = coefficient parameter of each of the three solvent mixtures (non-linear terms); and X_i_ = proportion of the components expressed in a 0.00–1.00 range. The special cubic model was used to add terms involving products of three components. Statgraphics 18^®^ software (Copyright 1982–1917 by Statgraphics Technologies, Inc., Rockville, MD, USA) was employed [33].

## 3. Results and Discussion

### 3.1. Assessment of Moisture and Lipid Values of Initial By-Products

The initial octopus by-products showed levels of 774.1 ± 0.3 and 31.4 ± 1.4 g·kg^−1^ for moisture and total lipids, respectively. Comparison of such values with edible parts of marine species in general indicate that the composition of the current by-products would correspond to a medium-fat fish species [34,35].

Compared to previous studies on cephalopod by-products, current moisture values were shown to be higher than those obtained for Argentinean shortfin squid (*Illex argentinus*) by-products (647.3 ± 4.4 g·kg^−1^) [36] but lower than in Patagonian squid (*D. gahi*) by-products (842.6 ± 2.2 and 859.3 ± 3.9 g·kg^−1^) [16,17]. Additionally, present moisture values were shown to be higher than those obtained in a wide range of studies focused only on particular viscera corresponding to different cephalopod species, such as octopus (*O. vulgaris*) digestive gland (62.5–64.0 g·100 g^−1^) and ovary (61.0–68.4 g·100 g^−1^) [22], giant squid (*Dosidicus gigas*) viscera (706.3 ± 1.3 g·kg^−1^) [37], or mitre squid (*Loligo formosana*) ovary (72.1 ± 0.2 g·100 g^−1^) [38].

According to the general inverse relationship between moisture and lipid values in marine species in general [34], comparison of the lipid content of the present by-products to previous related research showed opposite conclusions than those previously mentioned for the moisture value. Thus, current lipid values were higher than in Patagonian squid (*D. gahi*) by-products (19.0 ± 0.5 and 15.5 ± 0.1 g·kg^−1^) [16,17] but lower than in Argentinean shortfin squid (*I. argentinus*) by-products (111.3 ± 6.3 g·kg^−1^) [36]. Furthermore, present lipid levels were lower than those obtained in the octopus (*O. vulgaris*) digestive gland (5.4–9.0 g·100 g^−1^) [22] and in the giant squid (*D. gigas*) viscera (199.8 g·kg^−1^) [37].

In agreement with a previous study [16], the above-mentioned lyophilization process was necessary to increase the lipid yield by using the present low-toxicity solvents. This procedure, moisture elimination in general, has already been reported as necessary in previous research with relatively polar solvents such as ethanol and acetone [13,39].

### 3.2. Lipid Content of Lyophilized By-Products

Table 2 shows the lipid yield obtained for lyophilized octopus by-products by employing conventional and different kinds of low-toxicity solvents. Thus, the highest lipid value was obtained with the conventional procedure. Among the different low-toxicity systems checked, higher lipid values were obtained in solvent mixtures that included ethanol. Comparison to the conventional procedure showed that the highest recoverability values (*p* < 0.05) were obtained with ethanol/ethyl acetate and ethanol/acetone systems (ca. 80 and 79%, respectively). When employed alone, the three solvents led to the following increasing lipid recovery sequence: ethanol < acetone < ethyl acetate (ca. 55, 58, and 61% yield when compared to the conventional system, respectively).

Results regarding the extracting ability of the different systems tested can be explained according to their different physicochemical properties (polarity degree and others) and the kind of lipid substrate to be extracted [18,19]. A lower lipid extractability of low-toxicity systems than in the case of the conventional procedure can be explained on the basis that low-toxicity solvents tested are more polar than the chloroform/methanol mixture and, therefore, complete extraction of non-polar lipid classes like triacylglycerols (TAGs), waxes, or cholesterol esters, would be difficult. Remarkably, higher average recoveries were detected in the present work by employing extracting systems that include solvent combinations instead of single solvents.

Previous research also accounts for the extraction of the lipid fraction from marine biomass by low-toxicity solvents. Gigliotti et al. [13] tested the lipid fraction extraction of a polar-rich lipid substrate (Antarctic krill, *E. superba*) with different acetone/ethanol ratios. An increasing ethanol content in the extracting system led to a lipid yield increase so that higher lipid yields (g·100 g^−1^ dry krill) were obtained with solvent ratios (*v*/*v*) of 1/30 (ca. 13.) and 1/12 (ca. 12.0) than with 1/9 (ca. 9.5) and 1/6 (ca. 7.5) ratios. Furthermore, subcritical conditions for lipid extraction with hexane/isopropanol (3:2, *v*/*v*) from the microalga *S. obliquus* were employed [14]; the optimized total lipid yield was obtained by using 85 °C and 1.5 MPa conditions and led to an 82.6% recovery when compared to the conventional procedure. Recently, Li et al. [15] developed a comparative lipid extraction of the microalga *S. dimorphus* with different extracting systems (ethanol/hexane, 3:2; ethyl acetate/hexane, 1:1; methanol/hexane, 1:0.8; hexane; aq. 95% ethanol); in agreement with the present study, the highest lipid yields were observed with 95% ethanol. Recently, the same low-toxicity solvents as in the present study were tested on squid (*D. gahi*) by-products [16,17]; according to the present results, extracting mixtures that included ethanol led to higher lipid yields, and an increasing content of this solvent in the extracting system provoked a lipid yield increase.

Based on the interest on TL extraction by low-toxicity solvents and according to the present experimental design, the variance analysis by multiple regression of data corresponding to the TL content in the extracting solvents was carried out. Thus, the quadratic model was shown to be significant (*p* < 0.05) (Table 3). According to the variance analysis, the fitted model explained 96.55% of the variability of the TL content (R-square value) and the adjusted R-square value was 94.39%. Additionally, the standard error of the estimate (SEE) showed that the standard deviation of the residuals was 3.3108, this value indicating, approximately, the prediction errors (residuals) that can be taken into account in the current dataset.

**Figure 1:**
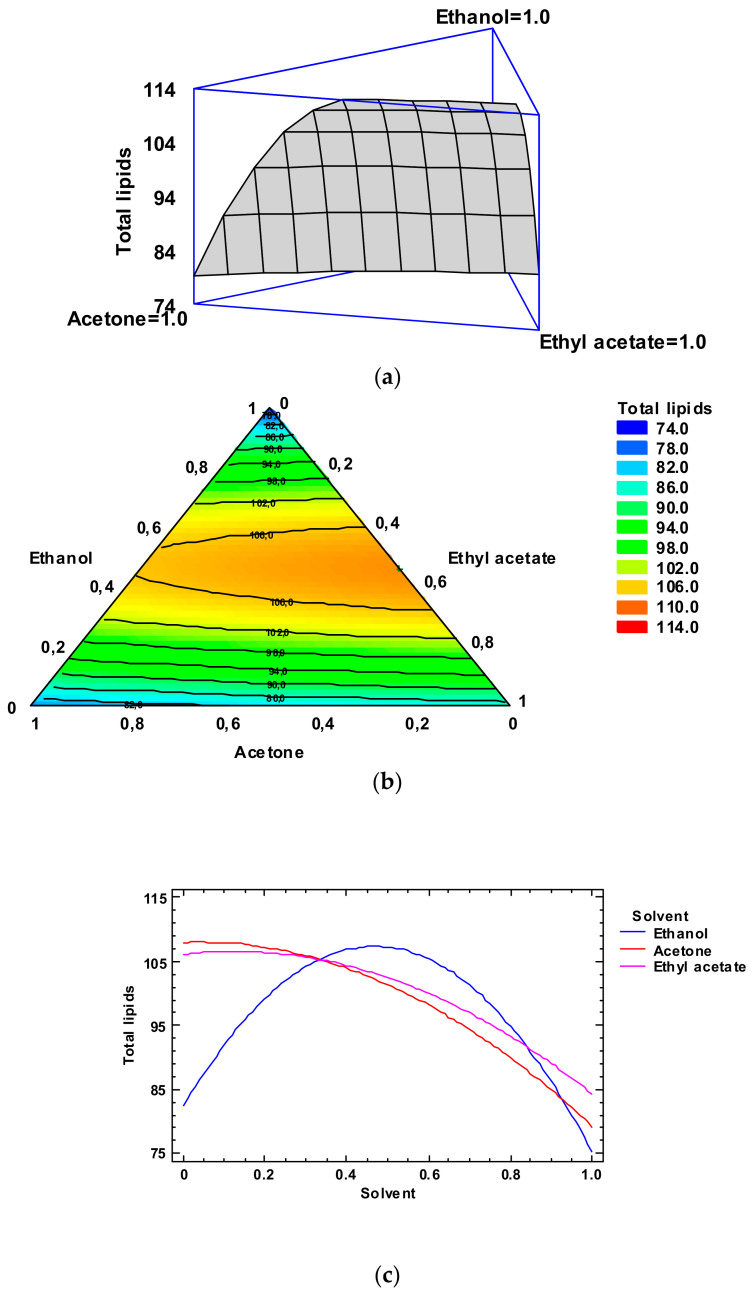
Application of the Simplex-Lattice design on the fitting model of the total lipid yields (g·kg^−1^ dry by-products) for lipid extraction of lyophilized octopus by-products. Panel (**a**): Estimated response surface; Panel (**b**): Contours of estimated response surface; Panel (**c**): Trace plot for lipid content.

In agreement with the Simplex-Lattice mixture design, the fitted model for TL value led to the estimated response surface depicted in Figure 1, Panel a. In it, the three experimental factors are the solvent concentrations, and the height of the surface denotes the TL content. An empirical coded equation could be employed to model the effect of the low-toxicity solvent concentrations. Thus, the following equation (Equation (2)) describes the fitted model that represents the estimated response surface of the TL content (g·kg^−1^ dry by-products) as a function of ethanol (E), acetone (A), and ethyl acetate (EA) relative concentrations in the extracting system (0.00–1.00 range):

TL content = 75.2711 × E + 79.2711 × A + 84.3211 × EA + 115.757 × E × A + 113.057 × E × EA + 2.6572 × A × EA(2)

In Figure 1, Panel b, the contour response surface of the estimated response surface for the TL content is described as a function of the low-toxicity solvent concentrations. The design is shown with a triangle where the three vertices represent the three pure solvents and the points placed on the triangle sides correspond to the binary mixtures. The solvent minimum level is 0.00 and the maximum is 1.00, which corresponds to pure solvent in coded variables. Each contour line represents combinations of low-toxicity solvent concentrations, which provide a selected value for the TL yield. Based on the optimization of the independent variables, the combination of factor levels which maximizes the TL content over the indicated region is 0.46/0.00/0.54 (ethanol/acetone/ethyl acetate, respectively). This combination would lead to an optimized value of 113.8 g·kg^−1^ dry by-products, which corresponds to a ca. 83% of the yield obtained by the conventional extraction. Comparison to TL values described in Table 2 shows that the most similar level was obtained by employing ethanol/ethyl acetate (0.50:0.50) as the extracting system (109.8 g·kg^−1^ dry by-products). Among the different experimental systems tested, this extracting mixture can be considered as the most similar to the above-mentioned optimized combination.

The trace plot for the lipid content is depicted in Figure 1, Panel c. This plot shows the effect on the TL content of a content increase or decrease in any of the relative concentrations of the solvents employed. Considering the centroid point (i.e., 0.33 value for each solvent), the TL content would increase as the ethanol and ethyl acetate concentrations increased and would decrease with increasing acetone concentration.

### 3.3. Phospholipid Content of Initial and Lyophilized By-Products

PL content of initial octopus by-products was 288.5 ± 22.2 g·kg^−1^ lipids. Comparison to related marine by-products shows that current values were lower than those obtained for Patagonian squid (*D. gahi*) by-products (450.8 g·kg^−1^ lipids) [16] and giant squid (*D. gigas*) viscera (468.7 g·100 g^−1^ lipids) [37]. Additionally, Głowacz-Rozynska et al. [40] found lower PL values in salmon (*Salmo salar*) skins (0.13 g·100 g^−1^ lipids), backbones (0.29 g·100 g^−1^ lipids), and heads (0.02–1.47 g·100 g^−1^ lipids). Compared to other marine substrates, the present PL level in the lipid fraction was found to be similar to the one reported in Antarctic krill (*E. superba*) (21–33 g·100 g^−1^ lipids) [13] and in central edible parts of megrim (*Lepidorhombus whiffiagonis*) muscle (290–329 g·kg^−1^ lipids) [29]. On the contrary, a lower PL content was detected in the megrim (*L. whiffiagonis*) edge muscle (37–70 g·kg^−1^ lipids) [29].

Table 2 shows the PL yield obtained for lyophilized octopus by-products by employing conventional and low-toxicity extracting systems. As for the TL determination, the conventional procedure also led to the highest PL values. Among low-toxicity solvents, the highest proportion of PLs was detected in the lipid fraction obtained by employing ethanol alone. A ca. 94% recovery was obtained when compared to the value obtained with the conventional procedure. Furthermore, high recovery values were detected in extracting systems with ethanol in the solvent mixture. In such cases, recoveries were included in the 65–70% range. Conversely, very low PL proportions were detected in lipid extracts obtained with systems that did not include ethanol.

The results can be explained on the basis of the physicochemical properties of the low-toxicity solvents tested and the nature of the PL substrate to be extracted According to the increasing relative polarity sequence [19] of ethyl acetate < acetone < ethanol, ethanol has been shown to extract a greater PL content than acetone or ethyl acetate; additionally, relatively non-polar lipid classes (i.e., TAGs, waxes, etc.) would not be completely extracted by ethanol so that the relative proportion of PLs would increase.

According to the present results, ethanol also showed a higher PL yield from squid (*D. gahi*) by-products than with acetone or ethyl acetate as extracting systems [16]. Contrary to the present results, a higher PL yield (ca. 30%) was obtained by applying a 1/6 ratio (acetone/ethanol) than in the case of employing a higher presence of ethanol (1/9, 1/12, and 1/30, acetone/ethanol; ca. 21–22%) during the extraction of PLs from krill (*E. superba*) [13]. In order to extract high levels of polar-rich lipid fractions from microalgal biomass, Jiménez Callejón et al. [39] proposed a two-step extraction; for it, a hexane extraction of neutral lipids was followed by an ethanol extraction of the resulting pellet, this consisting of a lipid extract including high levels of PLs and glycolipids.

PL classes are reported to be important constituents of cell membranes in general and therefore play a decisive role in living bodies. According to their amphiphilic character, PL molecules have been reported recently to be better vectors of ω3 PUFA compounds than other lipid classes like TAGs, methyl esters, or waxes [41,42]. Therefore, profitable functions related to the pharmaceutical and food production industries have been pointed out for marine PL compounds. These higher bioavailability and absorption properties have been shown to be especially relevant in ameliorating the triad of inflammation, oxidative stress, and immune cell aging, which represent common entities in aging and chronic diseases [43,44]. Additionally, antioxidant properties have been attributed to PL compounds during food processing based on the amine composition of their head group and on their FA composition [45,46].

In agreement with the interest in PL compound extraction with low-toxicity solvents, and according to the present experimental design, the variance analysis by multiple regression of data corresponding to the PL content in lipid extracts from low-toxicity solvents was carried out. Thus, the quadratic model was shown to be significant (*p* < 0.05) (Table 3). The variance analysis proved that the fitted model explained 98.97% of the variability of the total PL content (R-square value) and the adjusted R-square value was 98.32%. Additionally, the standard error of the estimate (SEE) indicated that the standard deviation of the residuals was 11.4246, this value indicating approximately the prediction errors (residuals) that can be taken into account in the current dataset.

**Figure 2:**
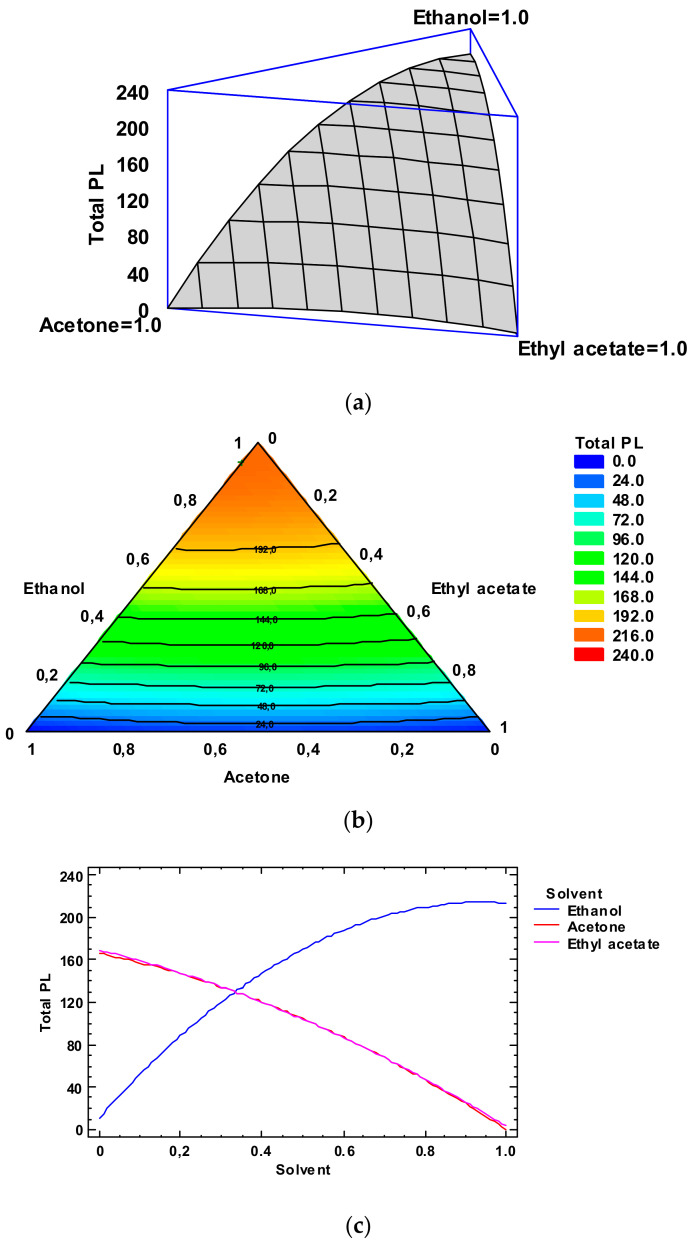
Application of the Simplex-Lattice design on the fitting model of the total phospholipid (PL) content (g·kg^−1^ lipids) of lyophilized octopus by-products. Panel (**a**): Estimated response surface; Panel (**b**): Contours of estimated response surface; Panel (**c**): Trace plot for PL content.

On the basis of the Simplex-Lattice mixture design, the fitted model for the PL value provided the estimated response surface indicated in Figure 2, Panel a. In it, the three experimental factors are the solvent concentrations, and the height of the surface denotes the PL content. An empirical coded equation could be employed to model the effect of the low-toxicity solvent concentrations. Thus, the following equation (Equation (3)) denotes the fitted model that represents the estimated response surface of the PL value (g·kg^−1^ lipids) as a function of ethanol (E), acetone (A), and ethyl acetate (EA) relative concentrations in the extracting system (0.00–1.00 range):

Total PL content = 212.852 × E + 0.151784 × A + 2.85178 × EA + 249.458 × E × A + 232.058 × E × EA + 39.2582 × A × EA(3)

In Figure 2, Panel b, the contour response surface of the estimated response surface for the PL value as a function of the low-toxicity solvent concentrations is depicted. The design is described with a triangle where the three vertices denote the three pure solvents and the points placed on the triangle sides denote the binary mixtures. The solvent minimum level is 0.00 and the maximum is 1.00, which corresponds to pure solvent in coded variables. Each contour line denotes combinations of the three low-toxicity solvent concentrations, which provide a selected value for the PL content. Based on the optimization of the independent variables, the combination of factor levels which maximizes the PL content over the indicated region is 0.93/0.07/0.00 (ethanol/acetone/ethyl acetate, respectively). This combination would lead to an optimized value of 217.3 g·kg^−1^ lipids, which corresponds to a ca. 95% of the yield obtained with the conventional procedure. Comparison to the PL values described in Table 2 shows that the most similar level was obtained by employing ethanol as the extracting system (214.8 g·kg^−1^ lipids). Among the different experimental systems tested, this extracting mixture can be considered as the most similar to the above-mentioned optimized combination.

Figure 2, Panel c includes the trace plot for the lipid content. This plot describes the effect on the PL content of a content increase or decrease in any of the relative concentrations of the solvents employed. Considering the centroid point (i.e., 0.33 value for each solvent), the PL content would increase as the ethanol concentration increases in the extracting system and would decrease as the acetone and ethyl acetate concentrations increased.

### 3.4. EPA and DHA Content of Initial and Lyophilized By-Products

The FA profile of the initial by-products obtained by the conventional procedure is indicated in Table S1. Thus, DHA was shown to be the most abundant FA followed by C16:0, EPA, C20:4ω6, and C18:0. The present FA profile is very similar to that of the edible parts of cephalopod species and marine species in general, which show a remarkable presence of PUFAs [22,29,34]. A similar profile was also detected in different kinds of by-products resulting from cephalopod species. Thus, DHA, C16:0, and EPA were shown to be the major FAs in Patagonian squid (*D. gahi*) [16] and in Argentinian shortfin squid (*I. argentinus*) [36] whole by-products. Additionally, this FA profile has also been detected in different single by-product fractions, such as liver and gonad of arrow squid (*Loligo bleekeri*) [47], digestive gland and ovary of octopus (*O. vulgaris*) [22], and squid (*Loligo formasana*) ovary [38]. Conversely, Shen et al. [48] found that MUFAs were predominant (C18:1ω9 and C20:1ω11) in cuttlefish (*Sepiella maindroni de Rochebrun*) viscera, and Kacem et al. [49] detected STFAs (C16:0 and C18:0) as the most abundant FA group in squid *(D. gigas*) viscera.

Among ω3 PUFAs, EPA and DHA values have received great interest on the basis of their beneficial health properties. Notably, clinical and epidemiological research has associated EPA consumption with low prevalence of circulatory, coronary, and inflammatory diseases [50], while DHA has been associated with the prevention of neurodegenerative diseases, fetal development, and correct functioning of the nervous system and visual organs in the fetus [51]. Therefore, the current research on individual FA composition will be focused on both PUFA compounds.

Table 2 shows the EPA content in the lipid extracts corresponding to the conventional and low-toxicity procedures applied to lyophilized octopus by-products. In all cases, EPA values were included in a narrow range (14.00–14.45 g·100 g^−1^ total Fas). Compared to the conventional extraction, a higher EPA content (*p* < 0.05) was detected in the lipid extract obtained by employing ethanol as the extracting system. Otherwise, no significant differences (*p* > 0.05) were obtained for other low-toxicity solvents when compared to the conventional procedure.

Concerning DHA, values obtained can also be considered to be included in a relatively narrow range (20.96–22.39 g·100 g^−1^ total Fas) (Table 2). The highest average value was obtained in the conventional procedure but was not significantly different (*p* > 0.05) when compared to the results obtained with ethanol as the extracting system. Conversely, all other low-toxicity extracting systems led to lower (*p* < 0.05) DHA levels than the conventional procedure. The lowest DHA content (*p* < 0.05) was found in lipid extracts obtained with extracting systems that did not include ethanol.

Previous studies have addressed the EPA and DHA values corresponding to lipid extracts of by-products obtained with different green extracting methods. Compared to the present values, higher contents of DHA (25.5 g·100 g^−1^ total Fas) were observed in precooked and non-precooked skipjack tuna (*Katsuwonus pelamis*) heads by Chantachum et al. [52] during comparative research on crude oil extraction with a wet reduction method; however, negligible levels of EPA were detected in both kinds of tuna samples. Lower values for EPA (7.1–7.4 g·100 g^−1^ total Fas) and DHA (8.0–8.3 g·100 g^−1^ total Fas) were observed in three different salmon (*Salmo salar*) by-products (backbones, skins, and heads) when extracted with several eco-friendly procedures (95 °C for 30 min, <15 °C, and enzyme-assisted) followed by pressing and centrifugation [40]. Furthermore, lower EPA (9.3 g·100 g^−1^ total Fas) and DHA (16.4 g·100 g^−1^ total Fas) values than in the current research were obtained from squid (*I. argentinus*) viscera with a wet pressing extraction [34]. Previous research tested the FA composition of the lipid extract obtained with ethanol, acetone, and ethyl acetate from squid (*D. gahi*) by-products [16]; contrary to the present results, EPA content was found to be higher in lipid extracts corresponding to ethanol-including and conventional extracting mixtures, and no effect of the extracting system was determined for the DHA presence in the lipid extract.

According to the high interest in the EPA and DHA contents in seafood and food in general and on the employment of low-toxicity solvents, an optimization study of the extracting systems was developed in the present work. In agreement with the Simplex-Lattice design developed, the variance analysis by multiple regression of data corresponding to EPA and DHA contents was carried out. Table 3 shows the results of the fitting model for both Fas.

As for the TL and PL content analysis, the quadratic model was demonstrated to be significant (*p* < 0.05) for the DHA content. The variance analysis showed that the fitted model could explain 95.54% of the variability of the DHA content (R-square value). The adjusted R-squared statistic, which is more appropriate for comparing models with different numbers of independent variables, showed a 92.75% value. The SEE indicated that the standard deviation of the residuals was 0.1548. According to such SEE value, it can be assumed that the forecasts and predictions are accurate.

**Figure 3:**
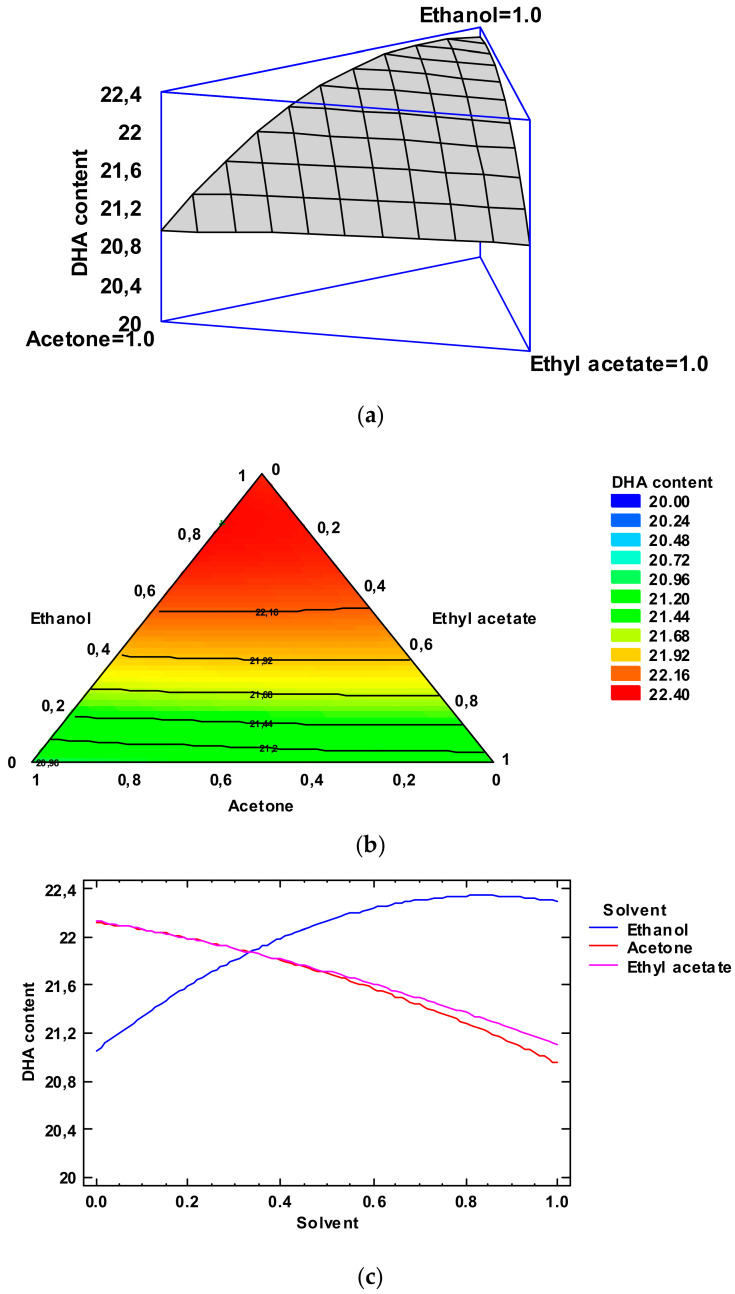
Application of the Simplex-Lattice design on the fitting model of the DHA content (g·100 g^−1^ total FAs) of lyophilized octopus by-products. Panel (**a**): Estimated response surface; Panel (**b**): Contours of estimated response surface; Panel (**c**): Trace plot for DHA content.

On the basis of the Simplex-Lattice mixture design, the fitted model for DHA content provided the estimated response surface depicted in Figure 3, Panel a. This figure shows the estimated DHA content as a function of ethanol, acetone, and ethyl acetate contents, while the height of the surface represents the DHA content. Remarkably, the effect of the low-toxicity solvent concentrations could be expressed with an empirical coded equation. Thus, the following equation (Equation (4)) indicates the fitted model that represents the estimated response surface of the DHA value (g·100 g^−1^ total FA) as a function of ethanol (E), acetone (A), and ethyl acetate (EA) concentrations (0.00–1.00 range):

DHA content = 22.2924 × E + 20.9474 × A + 21.1024 × EA + 2.04193 × E × A + 1.69193 × E × EA + 0.101934 × A × EA(4)

Figure 3, Panel b indicates the contours of the estimated response surface for the DHA values as a function of the low-toxicity solvent concentrations. Each contour line represents combinations of the three low-toxicity solvents, which give a selected value for the DHA content. In agreement with the optimization of the independent variables, the combination of factor levels which maximized the DHA content over the indicated region was 0.83/0.17/0.00 (ethanol/acetone/ethyl acetate, respectively). This combination would lead to an optimized value of 22.55 (g·100 g^−1^ total FA) for the DHA content; this value is higher than the average value obtained for the lipid extract obtained with the conventional method. A comparison with the DHA values described in Table 2 shows that the most similar level was obtained by employing ethanol as the extracting system (22.31 g·100 g^−1^ total Fas). Among the different experimental systems tested, ethanol alone can be considered as the most similar to the above-mentioned optimized combination. The FA profile of this extract is shown in Table S1.

Figure 3, Panel c indicates the trace plot for the DHA value. This plot indicates the effect on the DHA content of a content increase in any of the solvents checked. Taking into account the centroid point (i.e., 0.33 value for each solvent), the DHA content increased as the ethanol concentration increased in the extracting mixture and decreased with increasing acetone and ethyl acetate concentrations.

Concerning the EPA content, the Simplex-Lattice design was also applied. Thus, Table 3 shows the results of its fitting model. The model provided a *p*-value greater than 0.05 (i.e., 0.2003), which proved that the model is not statistically significant (*p* > 0.05). As a result, no subsequent variance analysis could be carried out as in the case of TL, PL, and DHA contents. Therefore, no optimization process of extracting conditions by employing the low-toxicity solvents included in the present study could be developed.

### 3.5. Determination of FA Groups and Ratios of Initial and Lyophilized By-Products

Based on the results obtained for the individual FA analysis, the following values were obtained for the FA groups (g·100 g^−1^ total Fas) in the lipid extract obtained with the conventional extraction of the initial by-products: 31.91 ± 0.16 (STFAs), 18.82 ± 0.30 (MUFAs), 49.27 ± 0.16 (PUFAs), 36.77 ± 0.19 (total ω3 Fas), and 12.50 ± 0.03 (total ω6 Fas). It can be concluded that the PUFA group is the most abundant in the lipid fraction of the current by-products. This FA group includes a very high proportion (ca. 75%) of ω3 Fas. Conversely, the presence of MUFAs was revealed to be markedly low. A similar FA group distribution to the present case was described for Patagonian squid (*D. gahi*) by-products [16]. Conversely, squid (*I. argentinus*) viscera showed a higher MUFA presence than in the present case [36].

Recent research has given great attention on certain FA group ratios according to several valuable properties. One of them is the total PUFA/total STFA ratio included in the human diet according to its direct relationship with digestibility, nutritional content, and preservative properties (i.e., antioxidant and anti-inflammatory) [53,54]. Additionally, seafood technologists found this ratio to be a valuable index for the lipid damage assessment and, accordingly, for the determination of the quality loss of seafood during different steps of processing [36,55]. The ratio value detected in the conventional extract of the present by-products (i.e., 1.54 ± 0.00) can be considered similar and, therefore, highly valuable when compared to lipid extracts corresponding to edible parts of different kinds of seafood [29,56].

Concerning the lipid extracts obtained with low-toxicity solvents, the values obtained for the PUFA/STFA ratio are included in a narrow range, i.e., 1.65–1.71 (Table 2). None of the low-toxicity solvent extracting systems showed significant differences *(p* > 0.05) with the conventional procedure. The highest average value was detected in the ethyl acetate extract, and it was found to be higher (*p* < 0.05) than the one corresponding to ethanol.

In the meantime, relevant interest has also developed in last years towards the ω3/ω6 FA ratio [57,58]. Remarkably, it has been demonstrated that most Western populations do not consume adequate quantities of ω3 Fas through natural sources. With the aim of preventing health concerns such as cardiovascular, neurological, and inflammatory disorders, the European Nutritional Society indicated that a human diet including an ω3/ω6 ratio of 1:5 or higher would lead to health benefits [59]. Additionally, the World Health Organization (WHO) recommends that this ratio should not be below 1:10 in the human diet [60]. Based on the ratio value obtained in the present case by the conventional procedure (2.94 ± 0.02), it can be concluded that current marine by-products include a highly healthy ω3/ω6 ratio.

Concerning the ω3/ω6 ratio values of the lipid extracts obtained with the low-toxicity solvents, the values can also be considered to be included in a narrow range (3.26–3.62; Table 2). Remarkably, extracts corresponding to all low-toxicity extracting mixtures provided higher (*p* < 0.05) ω3/ω6 values than the conventional procedure. The highest levels (*p* < 0.05) were obtained in lipid extracts consisting of acetone, acetone/ethyl acetate, and ethyl acetate extracting systems.

The comparison of the present ω3/ω6 FA ratios obtained in octopus by-products can lead to different and opposite conclusions with other marine substrates. The present ratio was shown to be lower than in the edible tissues of wild fish species such as megrim (*L. whiffiagonis*) (7.6–11.5) [29], anchovy (*Engraulis encrasicholus*) (6.3–12.5), and sardine (*Sardina pilchardus*) (7.7–10.1) [56], but similar to edible tissues from Indian sardine (*S. longiceps*) (3.7–5.1) [61]. A comparison with other kinds of by-products showed that the present ones provided lower values than those obtained by Šimat et al. [58] in tuna (*Thunnus thynnus*) and sardine (*S. pilchardus*) by-products (6.0–10.0 range), by Rodríguez et al. [36] in squid (*Illex argentinus*) viscera (7.6–8.0 range), and by Aubourg et al. [16] in squid (*D. gahi*) by-products (11.0–14.0 range). Conversely, lower ω3/ω6 FA ratios than in the current study were obtained in sea bass (*Sparus aurata*) and seabream (*Dicentrarchus labrax*) by-products (0.0–2.0 range) [58].

According to the interest on the PUFA/STFA and ω3/ω6 ratios and on the basis of the Simplex-Lattice design developed in the current study, the variance analysis by multiple regression of data corresponding to both ratios was carried out. Table 3 shows the results of the fitting model in both cases.

Concerning the results obtained for the PUFA/STFA ratio, the Simplex-Lattice design showed that the fitting model provided a *p*-value greater than 0.05 (i.e., 0.2214), which proved that the model was not statistically significant (*p* > 0.05). As a result, no subsequent variance analysis could be carried out as in the case of TL, PL, and DHA contents in order to carry out an optimization process of extracting conditions by employing the low-toxicity solvents included in the present study.

In the case of the ω3/ω6 ratio values for low-toxicity solvents, the quadratic model proved to be significant (*p* < 0.05) (Table 3). The variance analysis showed that the fitted model explained 86.66% of the variability of the ω3/ω6 ratio (R-square value), and the adjusted R-square value was 78.32%. Additionally, the SEE indicated that the standard deviation of the residuals was 0.0464. According to such SEE value, it can be assumed that forecasts and predictions are accurate.

**Figure 4:**
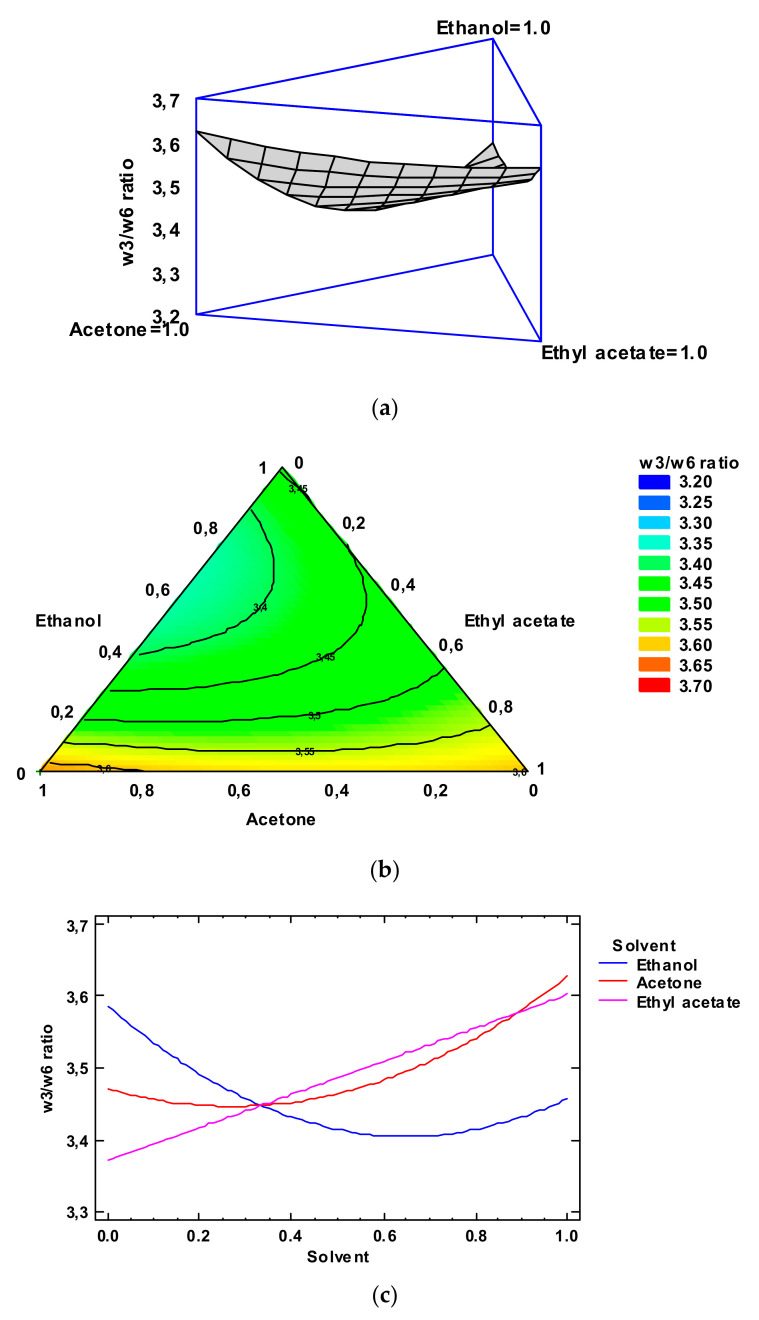
Application of the Simplex-Lattice design on the fitting model of the ω3/ω6 ratio of lyophilized octopus by-products. Panel (**a**): Estimated response surface; Panel (**b**): Contours of estimated response surface; Panel (**c**): Trace plot for ω3/ω6 ratio.

Based on the Simplex-Lattice mixture design, the fitted model for the ω3/ω6 ratio provided the estimated response surface depicted in Figure 4, Panel a. In it, the three experimental factors (ethanol, acetone, and ethyl acetate contents) are the low-toxicity solvent concentrations, and the height of the surface denotes the ω3/ω6 ratio value. The effect of the solvent concentrations could be described by an empirical coded equation. Thus, the following equation (Equation (5)) shows the fitted model that represents the estimated response surface of the ω3/ω6 ratio as a function of the ethanol (E), acetone (A), and ethyl acetate (EA) concentrations (0.00–1.00 range):

ω3/ω6 ratio = 3.45715 × E + 3.62715 × A + 3.60215 × EA − 0.683106 × E × A − 0.233106 × E × EA − 0.113106 × A × EA(5)

The contours of the estimated response surface for the ω3/ω6 ratio as a function of the solvent concentrations is indicated in Figure 4, Panel b. Each contour line represents combinations of the concentrations of the three low-toxicity solvents which lead to a selected value for the ω3/ω6 ratio. In agreement with the optimization of the independent variables, the combination of factor levels which maximizes the ω3/ω6 ratio over the indicated region is 0.64/0.00/0.36 (ethanol/acetone/ethyl acetate, respectively). This combination would lead to an optimized value of 3.70, higher than the average value obtained in the lipid extract corresponding to the conventional extracting procedure. A comparison to the ω3/ω6 ratio values described in Table 2 shows that the most similar level was obtained by employing ethanol/ethyl acetate (0.50:0.50) as the extracting system (3.48); among the different experimental systems tested, this extracting mixture can be considered to be the most similar to the above-mentioned optimized combination. The FA profile of this extract is shown in Table S1.

Figure 4, Panel c includes the trace plot for the ω3/ω6 ratio. This plot indicates the effect on the ω3/ω6 ratio values of a content increase or decrease in any of the solvents tested. On the basis of the centroid point (i.e., 0.33 value for each solvent), ω3/ω6 ratio values increased as the ethanol and ethyl acetate concentrations increased in the extracting mixture, while it decreased with increasing acetone concentration.

## 4. Conclusions

An optimization study of the extraction procedure with low-toxicity solvents (ethanol, acetone, and ethyl acetate) was performed on the recovery of valuable compounds from octopus (*O. vulgaris*) by-products. As a result, the Simplex-Lattice design developed indicated that the quadratic model was significant (*p* < 0.05) for the TL, PL, and DHA contents and for the ω3/ω6 ratio. Thus, the following optimized values were obtained: 113.8 g·kg^−1^ dry by-products (TLs), 217.3 g·kg^−1^ lipids (PLs), 22.55 g·100 g^−1^ total FAs (DHA), and 3.70 (ω3/ω6 ratio). According to the model developed, such optimized values correspond to the following relative solvent concentrations (ethanol/acetone/ethyl acetate): 0.46/0.00/0.54, 0.93/0.07/0.00, 0.83/0.17/0.00, and 0.64/0.00/0.36, respectively. Conversely, the results obtained for the EPA content and PUFA/STFA ratio did not provide a statistically significant (*p* > 0.05) quadratic model, so an optimized study could not be developed.

This study contributes to finding alternative sources for obtaining highly-valuable compounds from waste substrates resulting from seafood commercialization with the aim of providing an increased profitability to such biomass. Practical application would be facilitated by the fact that by-products are considered as a single product. However, according to the results obtained, a different solvent mixture would be necessary for each kind of valuable lipid compound. The suitability of low-toxicity solvents tested was determined and agrees with global technological interests in searching for alternative methods for oil extraction and for reducing the risk to humans and the environment of toxic chemical exposure. Based on an advanced mathematical design and on the employment of low-toxicity solvents, a novel strategy is proposed for the extraction, from octopus waste, of lipid compounds reported for their highly valuable effects on nutritional, health, and preserving properties. Before any subsequent use of octopus by-products, international requirements concerning safety aspects (presence of heavy metals, aromatic hydrocarbons, etc.) ought to be addressed.

## Figures and Tables

**Table 1:** Extraction conditions (from EC-1 to EC-14) and relative contents (0.00–1.00 range) of low-toxicity solvents for lipid extraction of lyophilized octopus by-products following the Simplex-Lattice mixture design.

Extracting Condition	Relative Content of Solvents (0–1 Range)		
	Ethanol	Acetone	Ethyl acetate
EC-1	1.00	0.00	0.00
EC-2	1.00	0.00	0.00
EC-3	0.50	0.50	0.00
EC-4	0.50	0.50	0.00
EC-5	0.50	0.00	0.50
EC-6	0.50	0.00	0.50
EC-7	0.00	1.00	0.00
EC-8	0.00	1.00	0.00
EC-9	0.00	0.50	0.50
EC-10	0.00	0.50	0.50
EC-11	0.00	0.00	1.00
EC-12	0.00	0.00	1.00
EC-13	0.33	0.33	0.33
EC-14	0.33	0.33	0.33

**Table 2:** Determination of TL, PL, EPA, and DHA values * and total PUFA/total STFA and total ω3/total ω6 ratios in different kinds of lipid extracts of octopus by-products **.

Extracting System	Response Variables					
	TL Content (g·kg^−1^ Dry By-Products)	PL Content (g·kg^−1^ Lipids)	EPA Content (g·100 g^−1^ Total FA)	DHA Content (g·100 g^−1^ Total FA)	PUFA/STFA Ratio	ω3/ω6 Ratio
Chloroform/Methanol (conventional procedure)	136.9 ± 8.3 f	228.8 ± 3.4 g	14.16 ± 0.16 ab	22.39 ± 0.09 d	1.66 ± 0.02 ab	3.26 ± 0.04 a
Ethanol	74.8 ± 0.4 a (54.6)	214.8 ± 2.3 f (93.9)	14.45 ± 0.00 c	22.31 ± 0.11 cd	1.65 ± 0.01 a	3.46 ± 0.02 c
Ethanol/Acetone (0.50/0.50)	107.9 ± 2.2 e (78.8)	161.1 ± 4.5 e (70,4)	14.18 ± 0.22 a	22.08 ± 0.01 b	1.69 ± 0.02 ab	3.38 ± 0.09 b
Ethanol/Ethyl acetate (0.50/0.50)	109.8 ± 3.8 e (80.2)	158.0 ± 1.9 e (69.1)	14.00 ± 0.02 a	22.07 ± 0.20 bc	1.65 ± 0.03 ab	3.48 ± 0.00 c
Acetone	78.9 ± 1.1 b (57.6)	2.1 ± 0.0 a (0.9)	14.32 ± 0.31 abc	20.96 ± 0.13 a	1.69 ± 0.04 ab	3.62 ± 0.01 d
Acetone/Ethyl acetate (0.50/0.50)	84.2 ± 1.4 c (61.5)	3.5 ± 0.0 b (1.5)	14.21 ± 0.01 b	21.00 ± 0.02 a	1.68 ± 0.02 ab	3.60 ± 0.05 d
Ethyl acetate	83.9 ± 2.7 c (61.3)	4.8 ± 0.0 c (2.1)	14.35 ± 0.31 abc	21.12 ± 0.09 a	1.71 ± 0.03 b	3.60 ± 0.01 d
Ethanol/Acetone/Ethyl acetate (0.33/0.33/0.33)	101.4 ± 3.2 d (74.1)	147.3 ± 2.2 d (64.4)	14.01 ± 0.04 a	21.97 ± 0.24 bc	1.65 ± 0.03 ab	3.42 ± 0.01 b

* Data expressed as average values ± standard deviations (*n* = 4). For TL and PL values, the percentage recoverability compared to the conventional procedure is indicated. In each column, average values followed by different lowercase letters (a–g) indicate significant differences (*p* < 0.05). ** Abbreviations employed: TL (total lipids), PL (phospholipids), EPA (eicosapentaenoic acid), DHA (docosahexaenoic acid), PUFA (polyunsaturated fatty acids), and STFA (saturated fatty acids).

**Table 3:** ANOVA application of the Simplex-Lattice mixture design of fitting model to response variables (TL, PL, EPA, and DHA contents; PUFA/STFA and ω3/ω6 ratios) obtained by applying low-toxicity solvents *.

Quadratic Model	Response Variables					
	TL	PL	EPA	DHA	PUFA/STFA	ω3/ω6
Model d.f.	6	6	6	6	6	6
*p*-value	0.0000	0.0000	0.2003	0.0001	0.2214	0.0038
Error d.f.	8	8	8	8	8	8
Standard error of the estimate	3.3108	11.4246	0.1734	0.1548	0.0265	0.0464
R-square (%)	96.55	98.97	58.52	95.54	57.12	86.66
Adjusted R-square (%)	94.39	98.32	32.59	92.75	30.32	78.32

* Abbreviations employed as expressed in Table 2.

## Data Availability

The data used to support the findings of this study can be made available by the corresponding author upon request.

## Supplementary Materials

The following supporting information can be downloaded at: https://www.mdpi.com/article/10.3390/foods12193631/s1. Table S1. Fatty acid (FA) profile (g·100 g^−1^ total FAs) of initial octopus by-products extracted with the conventional procedure and of lyophilised by-products extracted with ethanol and with ethanol/ethyl acetate (0.50:0.50)
