# Supplementary material for: Low-Toxicity Solvents for the Extraction of Valuable Lipid Compounds from Octopus (Octopus vulgaris) Waste
Source: Foods. 2023 Sep 30;12(19):3631. doi: 10.3390/foods12193631 (PMC10572350; doi:10.3390/foods12193631)
Supplement: Supplementary file 1 [file foods-12-03631-s001.zip › foods-2602592-supplementary.pdf]

Supplementary material

Table S1. Fatty acid (FA) profile (g·100 g^{-1} total FAs) of initial octopus by-products extracted with the conventional procedure and of lyophilised by-products extracted with ethanol and with ethanol/ethyl acetate (0.50:0.50)

FA	Initial by-products	Ethanol-extracted lyophilised by-products	Ethanol/ethyl acetate (0.50:0.50)-extracted lyophilised by-products
C14:0	3.00 ± 0.06	3.53 ± 0.04	3.48 ± 0.04
C15:0	0.73 ± 0.09	0.78 ± 0.06	0.85 ± 0.01
C16:0	17.77 ± 0.17	17.22 ± 0.06	16.84 ± 0.30
C16:1ω7	3.70 ± 0.07	5.17 ± 0.02	5.19 ± 0.05
C17:0	1.50 ± 0.04	1.50 ± 0.08	1.46 ± 0.01
C18:0	8.90 ± 0.18	6.95 ± 0.02	6.98 ± 0.00
C18:1ω9	6.81 ± 0.10	7.28 ± 0.03	7.60 ± 0.00
C18:1ω7	3.83 ± 0.13	4.49 ± 0.04	4.85 ± 0.03
C18:2ω6	0.75 ± 0.02	0.81 ± 0.02	0.83 ± 0.09
C20:1ω9	3.22 ± 0.04	2.75 ± 0.01	3.00 ± 0.02
C20:2ω6	0.89 ± 0.08	1.00 ± 0.00	1.11 ± 0.01
C20:4ω6	9.74 ± 0.05	8.37 ± 0.10	8.02 ± 0.13
C22:1ω9	0.85 ± 0.04	0.66 ± 0.01	0.68 ± 0.02
C20:5ω3	12.89 ± 0.11	14.45 ± 0.00	14.00 ± 0.02
C22:4ω6	1.12 ± 0.01	0.91 ± 0.01	0.93 ± 0.05
C24:1ω9	0.40 ± 0.02	0.27 ± 0.00	0.26 ± 0.02
C22:5ω3	1.63 ± 0.10	1.57 ± 0.03	1.84 ± 0.05
C22:6ω3	22.25 ± 0.08	22.31 ± 0.11	22.07 ± 0.22
